# Same-day endoscopic ultrasound, retrograde cholangiopancreatography and stone extraction, followed by cholecystectomy: A case report and literature review

**DOI:** 10.1016/j.ijscr.2020.04.063

**Published:** 2020-05-11

**Authors:** Eric Bergeron, Etienne Desilets, Thibaut Maniere, Michael Bensoussan

**Affiliations:** Departments of Surgery and Gastroenterology, Charles-LeMoyne Hospital, Greenfield Park, Canada

**Keywords:** Cholelithiasis, Choledocholithiasis, Cholecystectomy, Cholangiopancreatography, Endoscopic ultrasound, Case report

## Abstract

**Introduction:**

Common bile duct stone-related events and their recurrence are frequent even after endoscopic retrograde cholangiopancreatography and stone extraction. Cholecystectomy should be carried out as soon as possible after the initial episode to prevent complications.

**Case presentation:**

We present a case of a patient who underwent endoscopic ultrasound, cholangiopancreatography with stone extraction and cholecystectomy on the same day.

**Discussion:**

After a common bile duct stone-related event, investigation, and stone extraction, cholecystectomy should be carried out as soon as possible to avoid high rates of recurrence and complications. Rise in health care costs nowadays mandate such an approach as a pre-emptive measure to prevent health complications while lowering health costs.

**Conclusion:**

Diagnosis of common bile duct stone followed by extraction with endoscopic cholangiopancreatography and cholecystectomy on the same day is feasible, safe, and has many potential advantages.

## Highlights

• The risk of recurrence or complications is high after a common bile duct stone related event.
• Cholecystectomy should be carried out soon after extraction of a common bile duct stone.
• Same-day investigation, endoscopic stone extraction and cholecystectomy is feasible and safe.

## 1 Introduction

In the era of laparoscopic cholecystectomy, management of common bile duct stones has evolved along with the development of imaging modalities (external ultrasound, endoscopic ultrasound, magnetic resonance cholangiopancreatography (MRCP)) as well as improved availability and quality of endoscopic techniques (endoscopic retrograde cholangiopancreatography (ERCP), endoscopic sphincterotomy, laparoscopic common bile duct exploration) [[1], [2], [3], [4], [5]].

Recurrence of biliary related events occurs in the range of 17%–45% [[6], [7], [8], [9], [10]] with readmission rates between 10% and 18% within 30 days [6,8,10]. Even with ERCP and sphincterotomy, recurrence of acute pancreatitis or biliary stone related events occurs in 7%–20% of the cases [7,[10], [11], [12]] with a non-negligible proportion of cases with multiple episodes [7,11]. As much as 18% recurrence within two weeks of ERCP has been reported [3].

ERCP remains the most valuable option to remove choledocholithiasis [2]. However, ERCP, which is a potentially risky procedure [7], may be unnecessary [3], and as such it does not protect against recurrence of acute pancreatitis or other biliary events [7,9,10]. Preoperative endoscopic ultrasound or MRCP may overcome this problem in patients with persistent clinical suspicion but insufficient evidence of stones on abdominal ultrasonography [4,13].

Efficiency and cost containment have become the mainstay of current healthcare system [1,2], and thus reducing delays for investigation and procedures allows significant decline in healthcare costs [1,3,4,6,14,15], without compromising the quality of care [[1], [2], [3],^14^,^16^]. In order to illustrate these assertions, we present a patient who underwent endoscopic ultrasound, ERCP and laparoscopic cholecystectomy on the same day and was discharged within 24 h. This work is reported in line with the SCARE criteria [17].

## 2 Case presentation

A 44-year old female patient presented to the triage emergency department in the evening of July 17th, 2019. She complained of a crampy epigastric pain. She was slightly jaundiced but afebrile. She was otherwise in good health. This was her first and only episode. Blood tests were ordered. She was discharged with ibuprofen and a follow-up visit was planned the day after.

The patient was evaluated by the emergency physician the next morning. The pain was still present at the epigastrium. On examination, she was not in distress. She was still slightly jaundiced. Her body temperature was 36.5 °C. Blood pressure was 159/85 and pulse rate 90. The abdomen was depressible, with light sensitivity at the epigastrium and no rebound tenderness. Bilirubin was 51 μmol/L (normal: 3–21 μmol/L), alkaline phosphatase 418 U/L (normal: 35–105 U/L), aspartate aminotransferase 167 U/L (normal: 5–35 U/L), alanine aminotransferase 241 U/L (normal: 5–35 U/L). Hemoglobin was 130 g/L and white blood cell count 11.7/mm^3^.

**Fig. 1:**
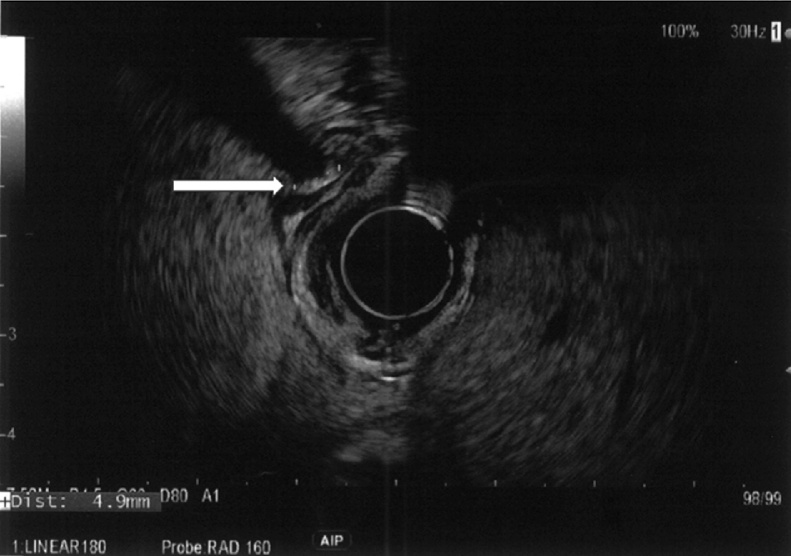
Endoscopic ultrasound showing a 4.9 mm gallstone and acoustic shadow (arrow) in the common bile duct.

A gastroenterology consultation was ordered. The gastroenterologist elected to go directly for an endoscopic ultrasound and considered that neither formal ultrasound nor tomodensitometry was necessary. This test was held at 10 AM and showed a dilated common bile duct at 10 mm with an impacted 4.9 mm stone (Fig. 1) and multiple stones in the gallbladder. The patient was brought for an ERCP, which was done few hours later at 1 P.M. Successful extraction and clearance of the common bile duct stone were performed.

Thereafter, the general surgeon on call was consulted and a laparoscopic cholecystectomy was scheduled. The cholecystectomy was carried out at 9 PM, when operating room was available. A 12 mm port at the umbilicus and two 5 mm ports at the right hypochondrium were introduced. The dissection was carried out with a bipolar hook. Cystic artery and duct were divided after clamping with Hem-o-lok™ clips. There was neither edema nor sign of inflammation. The procedure lasted 35 min without any difficulty or complication. The patient was discharged the day after in the morning. One month later, the patient showed an uneventful recovery.

## 3 Discussion

Guidelines have been previously published that recommend proceeding to cholecystectomy during the same admission after an episode of cholangitis [18] or acute pancreatitis [19]. Multiple studies demonstrated that cholecystectomy during index admission lowers recurrence of biliary related events and readmission rates [3,8,10,12,20], resulting in shorter length of hospital stay [3,14] and lower costs [6,15]. Despite recommendations [18,19], these guidelines are followed in less than 50% of the cases [5,8] for various reasons [6,9].

In the case presented here, endoscopic ultrasound, ERCP with stone extraction and laparoscopic cholecystectomy could have been done in the same day, in three different sessions. Performing three procedures on the same day (endoscopic ultrasound and ERCP, both done under sedation, followed by laparoscopic cholecystectomy) at three different times and in three separate wards is, to our knowledge, rarely reported. This remains a challenge for the patient and the physician. Even if there was no pancreatitis related to the common bile duct stone at the time of surgery, considerations about the risk of cholangitis, pancreatitis or recurrent common duct stones should be the same [6,8,10,13,19]. This management allowed the patient to be discharged the next day without difficulty.

Acute pancreatitis imposes a significant resource utilization with associated healthcare costs [8]. Gallstones pancreatitis represents more than 50% of all pancreatitis cases [5,16] and one of the most common emergency general surgery condition [6]. Mortality may attain 30% in severe pancreatitis [19] and more than 10% in severe cholangitis [14]. Further episodes of severe stone related events may thus be catastrophic and potentially lethal [19].

Even if cholecystectomy is planned within a short delay (less than one month), 30-day readmission rate for gallstone problems is between 10% and 21% [6,8,10]. Recurrence of acute pancreatitis may even reach as high as 18% within two weeks [3], with multiple episodes from 2.7%–14% of cases [7,11]. When cholecystectomy is not done, risk of recurrence may be as high as 45% [15].

ERCP with sphincterotomy diminishes but does not eliminate the incidence of recurrent pancreatitis [10]. Moreover, this procedure does not decrease the incidence of other biliary related events [10]. So, ERCP and sphincterotomy only mitigate the risk of recurrence of pancreatitis [9], cholecystitis and cholangitis [12,14,20] if cholecystectomy is not done [[10], [11], [12]] or done after long delay [10]. ERCP also carries inherent risk of early and late severe complications [7]. Gallstones with gallbladder left *in situ* is a significant risk factor for the recurrence [11] while cholecystectomy is significantly protective [7].

Mortality in patients with same-day cholecystectomy or same-admission cholecystectomy is zero or negligible [[1], [2], [3], [4],^8^,^10^,^16^]. Conversion rates of laparoscopy into laparotomy is not different in early versus delayed cholecystectomy in recent retrospective studies [1,3,6,21] or in randomized controlled trials [10,20]. Mortality is, however, higher in patients not undergoing cholecystectomy [13].

Although some authors prefer to avoid potential abdominal distension secondary to ERCP [4], others reported either absence or minimal distension during laparoscopy in their experience [1,3]. In the present case, abdominal dissection was as easy as for an “elective case” since there was neither adhesion nor edema. In few other studies, at worst, it was reported that in early cholecystectomy, there was edema and hyperemia without adhesions [16]. On the other hand, in delayed cholecystectomy, dissection may become more difficult [16,21].

Cholecystectomy is traditionally performed on a separate day after ERCP [2]. The performance of cholecystectomy within 24 h after ERCP has been proven to be safe [1,2,6]. Recent studies demonstrate the feasibility and security of combining ERCP and cholecystectomy on the same day [[1], [2], [3], [4], [5]] and even on the same anesthetic session [1,2,4], separately or simultaneously as a rendezvous technique [3,4]. In our case, combining ERCP and surgery at the same time was not possible because of logistic problems related to availability of equipment and supplies in an emergency situation.

The reported situation definitively represents an ideal one. The sequence of preoperative investigation and treatment for common bile duct clearance while waiting for the operating theater involves delays [2,4]. The management of early cholecystectomy after an episode of common bile duct stone event clearly necessitates logistic planning between gastroenterology and surgery services [1,2], and availability of local expertise and resources [6].

The cost saving is highly evident. It is nonetheless important that investigations and procedures can be securely achieved, even though healthcare cost is also a primary concern [[1], [2], [3], [4],^6^]. Studies concerning same-day procedures demonstrate further reduction in the length of stay and costs [[1], [2], [3], [4]], without increased risk of conversion to laparotomy or operative and postoperative complications [[1], [2], [3]]. However, considering the review of the literature and organizational concerns, we should recommend that, when facing a suspected or confirmed common bile duct stone, and if the condition of the patient allows, it is reasonable to rapidly actualize the investigation, followed by cholecystectomy as soon as possible, at worst within 72 h [5,8,10,16].

Same-day investigation, common bile duct stone extraction and cholecystectomy appear to be achievable and secure. Unfortunately, this ideal management is not always possible due to the conditions of the patients and logistic planning. On the other hand, delayed cholecystectomy entails significant risk of recurrence, risk of adverse events from subsequent ERCP and more difficult and complication-prone surgery, all these at increased healthcare costs [15].

## 4 Conclusions

Recurrence of common bile duct stone-related events is high even after a successful ERCP and stone extraction. Delaying cholecystectomy incurs significant risks of recurrence and adverse events from subsequent ERCP and late surgery.

After an episode of common bile duct stone and extraction, best results occur if cholecystectomy is done within 72 h and guidelines recommend cholecystectomy within the same admission. Same-day investigation, stone extraction and cholecystectomy can be securely achieved, minimizing healthcare costs, but is surely not always attainable.

Every effort should be directed to rapidly complete cholecystectomy after common bile duct stone extraction.

## Declaration of Competing Interest

None.

## Funding

EB will pay for the submission.

## Ethical approval

Ethical approval has been exempted by our institution.

## Consent

Written informed consent has been obtained from the patient.

In this paper, there is no possibility to identify the patient.

## Author contribution

EB and ED reviewed the record. ED, TM and MB commented on endoscopic procedure. EB commented on surgical procedure. All authors critically reviewed and approved the final version of the article.

## Registration of research studies

W/O.

## Guarantor

EB accept the responsibility for this work.

## Provenance and peer review

Not commissioned, externally peer-reviewed.
